# Desmoid Tumors in the Pediatric Population

**DOI:** 10.3390/cancers4010295

**Published:** 2012-03-09

**Authors:** Joshua N. Honeyman, Michael P. La Quaglia

**Affiliations:** Department of Surgery, Pediatrics Service, Memorial Sloan-Kettering Cancer Center, New York, NY 10065, USA; E-Mail: honeymaj@mskcc.org

**Keywords:** desmoid tumor, aggressive fibromatosis, surgery, pediatric surgical oncology

## Abstract

Desmoid tumors are benign soft tissue tumors associated with locally aggressive growth and high rates of morbidity, but they do not metastasize via lymphatic or hematogenous routes. While most of the data on desmoid tumors originates in the adult literature, many of the findings have been applied to the management of pediatric patients. This article discusses the epidemiology, etiology, clinical presentation, pathology, and treatment of this rare tumor in the pediatric population and includes a literature review of the most recent large series of pediatric patients with desmoid tumors.

## 1. Introduction

Desmoid tumors are rare soft tissue neoplasms that do not metastasize, but exhibit aggressive growth and local invasion. Thus, they are designated as “intermediate” tumors, according to the World Health Organization classification of soft tissue tumors [1]. Desmoid tumors, also called aggressive or deep-seated fibromatoses, affect all age groups and can occur throughout the body. In children, relatively few reports exist in the literature to provide guidance in the management of these complex patients, and multiple treatment plans have been proposed without much prospective evidence to validate one approach over another.

In this review of desmoid tumors in the pediatric population, we will first provide a general summary of the current data regarding desmoid tumors, including the surgical management of this complex disease. We will also specifically review the literature on the initial treatment and outcomes of children with desmoid tumor in recent large series of patients.

## 2. Epidemiology and Etiology

Desmoid tumors are uncommon in the general population, with an estimated annual overall incidence of 2 to 4 new cases per 1 million per year [2]. The peak incidence among pediatric patients occurs between 5 to 8 years of age, although these tumors can occur throughout childhood.

While the etiology of desmoid tumors remains unclear, some correlating factors have been well described. Tumors are commonly associated with dysregulation of the beta-catenin pathway. The tumor suppressor gene *APC* regulates cellular levels of beta-catenin, which in turn alters nuclear transcription and signaling in the Wnt pathway. Perturbation of this pathway is believed to result in deregulation of connective tissue growth and increased tumor cell proliferation. Approximately 12% to 25% of patients with inherited mutations in *APC* (familial adenomatous polyposis, Gardner’s syndrome) will eventually develop a desmoid tumor [3,4]. In contrast, sporadic desmoid tumors rarely have somatic mutations in *APC* [5]; direct mutations in beta-catenin are more common [6].

Twenty percent of patients diagnosed with desmoid tumor will have some history of antecedent trauma to the region [7], including surgical trauma. However, the causative role of trauma in desmoid tumors is not well characterized. Desmoid tumors occurring in patients with *APC* mutations have been noted to frequently occur shortly after a patient’s first abdominal surgery. Colectomy in patients younger than 18 years has also been associated with increased incidence of desmoid tumors in females [8].

Desmoid tumors have also been associated with high estrogenic states, including pregnancy, and studies have found female sex to be a risk factor for development of a desmoid tumor [2]. Immunohistochemical analysis has demonstrated estrogen receptors within tumors [9], and anti-estrogen treatment has been shown to restrict growth of desmoid cells *in vitro* [10], suggesting that cellular proliferation may be mediated via a hormonal pathway.

## 3. Clinical Presentation and Pathology

Typically, patients with desmoid tumors will present with a painless enlarging mass. However, the pattern of presentation is contingent upon the region in which the tumor develops. Symptoms can include pain, dysphagia, bowel obstruction, and limb dysfunction. Approximately 6% of patients will present with synchronous or metachronous multifocal disease [7,11].

Intra-abdominal desmoid tumors account for 5% to 7% of disease in children [12,13]. Arising from the intestinal wall or the mesentery, intra-abdominal tumors are often associated with *APC* mutations, and they carry the highest risk of mortality. Extra-abdominal desmoid tumors are found in the trunk, the extremities, and the head and neck. Over half of all pediatric desmoid tumors occur in the extremities [7]. Tumors arising in the head and neck account for 17% to 30% of all desmoid tumors [12,13,14], and 8% to 19% of tumors develop in the abdominal wall [12,13].

The diagnosis of desmoid tumor requires pathologic review of a tissue specimen. Core biopsies or open surgical biopsies are preferred to obtain sufficient tissue for detailed review of tissue structure as well as immunohistochemical analysis. Fine needle aspiration is typically insufficient to obtain a specific diagnosis [15].

**Figure 1:**
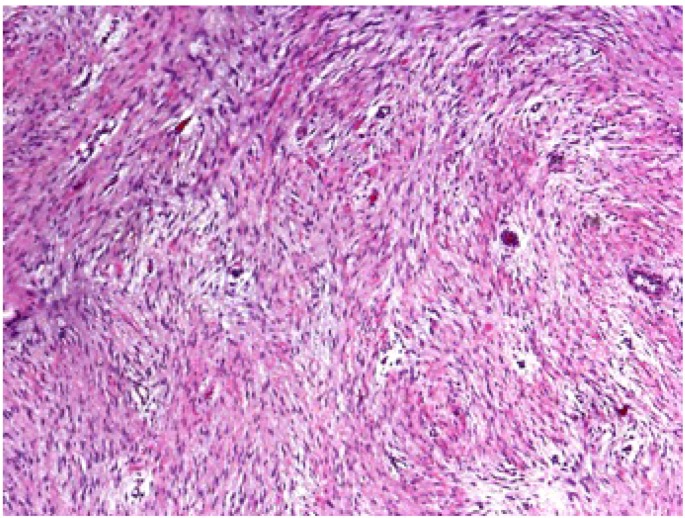
The microscopic appearance of a desmoid tumor reveals well-differentiated cells and bands of fibrous tissue.

Grossly, desmoid tumors are firm and non-encapsulated masses arising from regions of connective tissue. Margins can be poorly defined and difficult to identify. Microscopically, desmoid tumors are bland, appearing with well-differentiated cells and bands of fibrous tissue (Figure 1).

Immunohistochemical markers assist in the diagnosis of desmoid tumors, although there can be significant overlap in the immunohistochemical profile between a variety of soft tissue tumors [7,16]. Cells typically stain diffusely for vimentin, an intermediate filament expressed in mesenchymal cells, as well as for smooth muscle actin. Tumor cells demonstrate an absence of desmin, S-100, and CD34. While reports have demonstrated immunoreactivity for c-Kit [16], the majority of tumors are primarily c-Kit negative [17].

## 4. Imaging

The goals of radiographic assessment of desmoid tumors are to determine the precise anatomic location, including the tumor’s relationship to important regional structures, and to evaluate for extent of disease. These issues influence treatment planning, particularly during evaluation for surgical resection [18].

**Figure 2:**
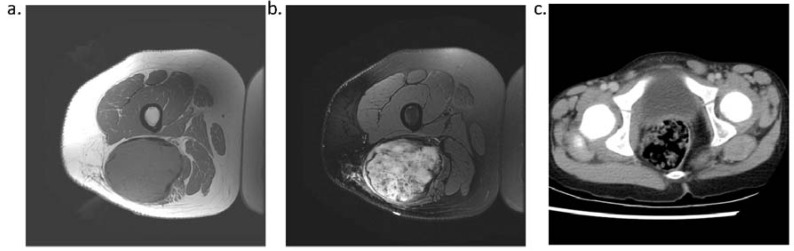
T1 (**a**) and T2 (**b**) weighted MRI and contrast-enhanced CT (**c**) of desmoid tumors provide anatomic information useful in the management of desmoid tumors.

Because desmoid tumors arise from the soft tissue, magnetic resonance imaging is the most appropriate imaging modality to assist with diagnosis and to evaluate margins and extent of disease (Figure 2). The appearance of these tumors can vary from hypo-intense to hyper-intense in comparison to muscle, and the signal is, overall, heterogeneous [19,20]. There is no standard enhancement pattern after injection of gadolinium.

On plain radiography, desmoid tumors appear as ill-defined masses or soft tissue swelling. CT scans can provide valuable anatomic information, particularly in regard to bone involvement [21].

## 5. Treatment

### 5.1. Surgery

Traditionally, gross total resection has been the mainstay of treatment for desmoid tumors. In children, recurrence rates after surgery are reported to range between 22% and 76% [12,13], and multiple large series have determined that a negative surgical margin is the strongest predictor of event-free survival [2,12,13].

Consequently, most patients will undergo early operative intervention in an attempt to obtain gross total resection of the tumor. Occasionally, these procedures occur at the expense of great morbidity and potential long-term disability. While this approach may provide the best opportunity for cure, pursuit of negative margins in patients may also increase morbidity without significantly impacting outcomes [22]. In addition, recurrence does not correlate with response to systemic therapy or survival [13].

Reassessment of this approach to the treatment of desmoid tumors first appeared in the literature on adult patients, in whom the indolent course of disease and the low risk of many desmoid tumors contrasted heavily with the high morbidity of certain surgical interventions [22,23]. In adult patients, a period of watchful waiting prior to committing to operative resection did not negatively impact outcome and, in fact, lowered overall morbidity [24]. This period of observation provides time to assess the natural history of the tumor, which may remain stable for a long period or may even regress. For those tumors that enlarge or cause significant symptoms, an operation would then be indicated. To date, no series has directly compared pediatric patients undergoing aggressive operative resection to patients treated with a period of close observation.

### 5.2. Adjuvant Treatment Options

Adjuvant treatment must be considered for patients with residual disease as well as in the setting of recurrent, progressive, or unresectable tumor. Chemotherapeutic choices include traditional cytotoxic therapies [25,26], and non-cytotoxic agents that exploit the endocrine and inflammatory pathways implicated in the pathogenesis of desmoid tumors [27].

Early reports of chemotherapy for desmoid tumor involve children with head and neck desmoid tumors treated with doxorubicin/dacarbazine [28] and vincristine/actinomycin/cyclophosphamide [29,30] combination regimens. A vinblastine (5 mg/m^2^/dose) and methotrexate (30 mg/m^2^/dose) combination, administered weekly for 26 weeks, then every other week for another 26 weeks, was tested in a phase 2 trial in children; the response rate was 31%, with stable disease in an additional 38% of patients [26]. However, 74% of patients experienced moderate to severe toxicity, and 19% had life-threatening toxicity. Adult and pediatric patients receiving combination chemotherapy have had overall response rates ranging from 17–100% with a median of 50% [31]. Doxorubicin-based therapy has demonstrated good response rates in adults [32], but treatment is limited by anthracycline-related toxicities.

The primary non-cytotoxic treatment options are nonsteroidal anti-inflammatory agents and hormonal agents. Anti-inflammatory drugs such as indomethacin, sulindac, and diclofenac have shown efficacy in desmoid tumors [33,34,35]. Initial interest in NSAIDs as a treatment option for desmoid tumors arose after a single case report of regression of a sternal desmoid tumor while the patient was receiving indomethacin for pericarditis [36].

These agents act via inhibition of prostaglandins, which may have immunomodulatory or vascular effects, or may be directly toxic to the tumor itself. A systematic review of trials and case series of adults treated with NSAIDs found a response rate of around 50%, although many responses took over 24 months to manifest [31].

Anti-estrogen therapy exhibits anti-proliferative activity in desmoid tumors [10]. Hormonal therapies including tamoxifen, toremifene, megestrol, progesterone, testolactone, and goserelin have shown efficacy in desmoid tumors [31]. Response rates of over 50% have been reported for endocrine treatments in adults [31,37,38]. While both anti-estrogen and anti-inflammatory drugs have been used in pediatric patients [39], their efficacy has not been fully proven. Furthermore, the long-term impact of hormone-based therapies on the growth and development of pediatric patients is unclear.

Although desmoid tumors are considered to be c-Kit negative [17], the tyrosine kinase inhibitor imatinib mesylate has also demonstrated some efficacy in the treatment of desmoid tumors [40,41]. Response rates in adults have ranged from 6% to 16% [40,42]. The mechanism of action for imatinib in desmoid tumor may be due to action on platelet-derived growth factor beta (PDGFRB) tyrosine kinase receptor, although the target molecule has not been definitively identified.

Other pharmacologic agents have been considered in the treatment of desmoid tumors in children, including hydroxyurea [43] and interferon alpha [44].

Adjuvant radiotherapy can help mitigate the negative effects of positive surgical margins [45,47]. A recent review of 30 pediatric and young adult patients found that younger age was associated with inferior locoregional control after radiotherapy [45]. Significantly, doses exceeding 55 Gy resulted in both improved tumor control as well as increased risk of complications.

Complication rates range between 5% and 40% and are heavily dependent on dose and field [45,47]. Radiation-associated complications in pediatric patients include growth disruption of the immature skeleton, fractures, soft-tissue injury (cellulitis, necrosis), and secondary malignancy. Thus, the benefits of radiotherapy in establishing local control of desmoid tumors must be weighed against the relative risks of both short- and long-term complications from the radiation exposure.

## 6. Literature Review

### 6.1. Methods

PubMed was queried for all articles matching “aggressive fibromatosis” OR “desmoid tumor” within the past 20 years. Limits applied to the search included “English language,” “Humans,” and “All Children (Age 0-18).” Search results included 349 matching articles. Titles and abstracts were evaluated to exclude review articles, case reports, and basic science reports. The remaining articles were reviewed, and papers were excluded that contained fewer than 10 patients, lacked survival data, dealt with desmoid tumor of only a single anatomic location, or did not contain information on the primary tumor and the initial course of treatment for patients in the study. A total of six patient series were identified; a study by Skapek *et al.* included two treatment groups, one with primary tumors and one with recurrent tumors [26]. Where available, data were abstracted regarding demographics, treatment, and outcome.

### 6.2. Results and Discussion

In total, the six studies included 219 patients with median ages ranging from 7 to 13 years. Gender distribution was relatively equal for the studies (103 males and 106 females). The anatomic distribution of tumors was 56 head and neck, 32 trunk, and 105 extremity. Four studies included patients with intra-abdominal tumors (n = 12). Multicentric tumors occurred in 9 patients (Table 1).

**Table 1:** Literature review: Demographics and tumor location.

Reference	Patients	Age (y)	Sex (M:F)	Tumor Location				
				Head and Neck	Trunk	Extremity	Intra-abdominal	Multicentric
Faulkner *et al*. [12]	63	NS	30:33	11	5	39	3	5
Spiegel *et al.* [48]	18	7	12:6	5	1	12	1	2
Buitendijk *et al.* [7]	13	4	6:7	5	4	4	0	1
Skapek *et al.* [26]	10	11	NS	NS	NS	NS	NS	NS
Jabbari *et al.* [49]	21	13	11:10	4	4	12	1	1
Meazza *et al.* [13]	94	10	44:50	31	18	38	7	NS

NS = not specified.

In the three earliest studies, all patients underwent surgical resection (gross-total or subtotal) as part of the initial treatment regimen [7,12,48] (Table 2); the most recent studies included patients who underwent initial non-operative treatment [13,26,49]. No study compared patients undergoing a period of observation only. The study by Meazza *et al*. included three groups: complete resections, gross total resections with microscopic margins, and biopsy-only patients plus patients with subtotal resections. The complete resection cohort had the best event-free survival (77.2% at 5 years), but the biopsy/subtotal resection group had better event-free survival than the group with microscopically positive margins (35.2% *versus* 28% at 5 years). A minority of patients received either chemotherapy or radiation as part of their initial treatment regimen [13] (Table 2).

There was a total of four deaths in the included studies. One patient died due to severe radiation-induced dermatitis and ulceration [12], one from intramedullary brainstem progression [48], and two patients died of tumor progression with abdominal disease [13].

While the treatment of desmoid tumors is evolving, most patients in the recent studies have experienced a recurrence in the years following diagnosis. Recurrence rates for these studies were well above 50%, with rates ranging between 60% and 80%.

**Table 2:** Literature review: Treatment and outcome.

Reference	Surgery	Margins		Chemotherapy	Radiation	Rate of Recurrence	Deaths	Median Follow-up
		+	−					
Faulkner *et al.* [12]	63 (100%)	NS	NS	6 (10%)	17 (27%)	65%	1	6 y
Spiegel *et al.* [48]	18 (100%)	15	3	5 (28%)	0 (0%)	83%	1	8 y, 4 mo
Buitendijk *et al.* [7]	13 (100%)	6	6	3 (23%)	2 (15%)	23%	0	3.9 y
Skapek *et al.* [26]	0 (0%)	NA	NA	10 (100%)	0 (0%)	40%	0	NS
Jabbari *et al.* [49]	17 (81%)	14	3	5 (24%)	11 (52%)	71%	0	75.7 mo
Meazza *et al.* [13]	78 (83%)	55	23	15 (15%)	6 (6%)	63%	2	130 mo

NA = not applicable; NS = not specified.

## 7. Conclusions

Relapse rates and morbidity for children with desmoid tumors remain high. Because of the low incidence of this disease, there are few large studies on desmoid fibromatosis in children. In those studies that have been published, there is broad variability in study population and in treatment regimen. Mounting evidence from the adult literature suggests that expectant management of desmoid tumors is possible in select patients, and that surgical intervention should be reserved for symptomatic tumors or rapidly progressing disease. While data obtained from adults can provide valuable insight into the pediatric disease, it is essential to further characterize the natural history, treatment effect, and outcomes in the pediatric population.

The treatment of children with desmoid tumor often requires a multidisciplinary approach by clinicians experienced with the management of pediatric cancer. For localized tumors that permit uncomplicated gross total resection, surgery is the first-line treatment. In other patients, particularly those in whom surgery may result in significant morbidity, a period of watchful waiting should be attempted. For the subset of patients who demonstrate rapid growth, pain, or functional compromise, an attempt at surgical resection should be made. Radiation and chemotherapy may assist in maintaining local control of desmoid tumors in patients with positive margins or residual disease.
